# Meta-analysis of the efficacy of Jingjin acupuncture therapy in the treatment of spastic cerebral palsy

**DOI:** 10.3389/fneur.2024.1358732

**Published:** 2024-05-09

**Authors:** Xingyu Kang, Ying Huang, Yi Zheng, Qian Zhang, Rui Gong, Jinlang Tan, Le Ma, Siyu Chen, Xueyan Lv, Shuai Shi

**Affiliations:** ^1^Graduate School, Heilongjiang University of Chinese Medicine, Harbin, China; ^2^Rehabilitation Department, The Second Affiliated Hospital of Heilongjiang University of Chinese Medicine, Harbin, Heilongjiang, China

**Keywords:** Jingjin, meridian, tendon, acupuncture, spasticity, cerebral palsy

## Abstract

**Background:**

This study aimed to systematically evaluate the clinical efficacy of Jingjin (muscle region of the meridian, sinew/tendon/fascia) acupuncture therapy for the treatment of spastic cerebral palsy.

**Methods:**

Computer searches of the Cochrane Library, Web of Science, PubMed, Embase, Chinese Biomedical Literature (CBM) Database, Wanfang database, Wipu (VIP) database, and China National Knowledge Infrastructure (CNKI) database for published randomized controlled trial (RCT) studies on Jingjin acupuncture treatment of cerebral palsy from the beginning of the database construction until 30 November 2023 were performed, and the quality of the papers was assessed through independent data extraction by two individuals and then meta-analyzed using RevMan5.4 software. A total of 20 RCTs involving 1,453 patients were included.

**Results:**

The overall effective rate of Jingjin acupuncture therapy was better than that of conventional therapy, with a combined odds ratio (OR) of 4.70 and a 95% confidence interval (CI) of [3.05, 7.24]. The Modified Ashworth Spasticity (MAS) Scale, Gross Motor Function Measure (GMFM), Fine Motor Function Measure (FMFM), and Comprehensive Spasticity Scale (CSS) scores are superior to conventional therapy.

**Conclusion:**

Jingjin acupuncture therapy is effective in treating spastic cerebral palsy and has better overall efficacy than conventional therapy. Due to the low quality of some of the literature in this study type, more high-quality, well-designed clinical studies are needed to validate it.

## 1 Introduction

Cerebral palsy (CP) is a static, non-progressive disorder caused by brain damage or injury during the prenatal, perinatal, and postnatal periods and is a major developmental disorder that affects children’s functioning (1). The motor deficits of CP are often accompanied by sensory, perceptual, cognitive, communication, behavioral disorders, seizures, and secondary musculoskeletal problems (2). According to epidemiologic surveys, the prevalence of CP in the world is 0.2–0.6% (3). It is the most common childhood physical disability, affecting 2–2.5 children per 1,000 births in Western countries (4, 5). Spasticity is the most common form of dystonia (6). CP can be classified according to functional ability and neurological subgroups based on the limb involved (hemiparesis, quadriplegia, or diplegia), clinical signs and symptoms (spasticity, dyskinesia, or ataxia), and muscle tone (hypotonia or hypertonia) (7). Although several classification systems are available, the Gross Motor Function Classification System-Extended and Revised (GMFCS-E&R) is the most commonly used system and consists of five levels, I (minimal impairment) to V (most severe impairment). This system provides a standardized classification that facilitates prognosis, treatment, and effective communication between clinicians, researchers, parents, and carers (8).

Dystonia abnormalities in children with CP are also the focus and difficulty of rehabilitation, and currently, rehabilitation training is the main treatment modality (9–11). Functional training can alleviate the condition to a certain extent, but the overall effect is not ideal. Jingjin (muscle region of meridian, sinew/tendon/fascia) acupuncture (12–15) as a Chinese medicine therapy, currently provides a new way of thinking for treating pediatric CP. Jingjin acupuncture therapy was first recorded in “Ling Shu - Jingjin,” and it refers to the treatment of meridian tendon disease by applying needles to the painful points of meridian tendons. Under the guidance of the Jingjin theory, the later generations have continuously invented and developed needles and needling methods, which has led to the significant progress of Jingjin needling therapy, which includes various kinds of needles (loose tendon needles, sharp-edged needles, water-needle knives, hooked-sharp needles, rounded sharp needles, etc.) and needling methods (rows of stabs, penetrating stabs, millimeter stabs, Qi stabs, Hegu stabs, Zhuang medicine picking stabs, etc.) (16). In recent years, many studies (17, 18) have indicated that Jingjin acupuncture can improve CP samples and high-quality clinical trials. This study aimed to evaluate the clinical efficacy of Jingjin acupuncture for treating dystonia in children with spastic CP through meta-analysis and to provide an evidence-based medical foundation for the clinical treatment of dystonia in children with spastic CP.

## 2 Materials and methods

### 2.1 Study design

This meta-analysis follows the guidelines provided in the Preferred Reporting Items for Systematic Reviews and Meta-Analysis (PRISMA) and proceeds based on the PICO principles for study design. P-Participants/population: Participants were eligible for clinical diagnosis of spastic CP without restriction of age, gender, or ethnicity. I - intervention(s): The study group was treated with acupuncture on meridian tendons based on the treatment given to the control group; C - Comparator (s)/control: The control group, which did not receive treatment of acupuncture on meridian tendons, was not limited to rehabilitation treatment modalities; O - Outcome (s): The main outcome indicators included the overall effectiveness, Modified Ashworth Spasticity (MAS) Scale, Gross Motor Function Measure (GMFM), Fine Motor Function Measure (FMFM), and Comprehensive Spasticity Scale (CSS).

### 2.2 Search strategy

An electronic search of databases, including MEDLINE/PubMed, Web of Science, the Cochrane Library, EMBASE, CBM, CNKI, WanFang, and VIP, was performed to collect randomized controlled clinical studies on the treatment of acupuncture on meridian tendons for CP from the beginning of the database construction until 31 October 2023. Medical Subject Headings (MeSH) and non-MeSH phrases were used, including acupuncture, meridian tendons, needle, needles and knives, prick, bladed needle, CP, spasticity, and their corresponding English keywords, and the search language was limited to Chinese and English. Taking the Pubmed database as an example, the detailed search strategy is shown in Table 1.

**Table 1:** PubMed search strategy.

No.	Search strategy
#1TS	(acupuncture) OR (meridian tendons) OR (needle) OR (needles and knives) OR (prick) OR (bladed needle)
#2TS	[CP (Cerebral Palsy)] OR (Cerebral Palsy, Dystonic-Rigid) OR (Cerebral Palsies, Dystonic-Rigid) OR (Cerebral Palsy, Dystonic Rigid) OR (Dystonic-Rigid Cerebral Palsies) OR (Dystonic-Rigid Cerebral Palsy) OR (Cerebral Palsy, Mixed) OR (Mixed Cerebral Palsies) OR (Mixed Cerebral Palsy) OR (Cerebral Palsy, Monoplegic, Infantile) OR (Monoplegic Infantile Cerebral Palsy) OR (Infantile Cerebral Palsy, Monoplegic) OR (Cerebral Palsy, Quadriplegic, Infantile) OR (Quadriplegic Infantile Cerebral Palsy) OR (Infantile Cerebral Palsy, Quadriplegic) OR (Cerebral Palsy, Rolandic Type) OR (Rolandic Type Cerebral Palsy) OR (Cerebral Palsy, Congenital) OR (Congenital Cerebral Palsy) OR (Little Disease) OR (Little’s Disease) OR (Spastic Diplegia) OR (Diplegias, Spastic) OR (Spastic Diplegias) OR (Diplegia, Spastic) OR (Monoplegic Cerebral Palsy) OR (Cerebral Palsies, Monoplegic) OR (Cerebral Palsy, Monoplegic) OR (Monoplegic Cerebral Palsies)
#3TS	(Spasticity, Muscle) OR (Spastic) OR (Clasp-Knife Spasticity) OR (Clasp Knife Spasticity) OR (Spasticity, Clasp-Knife)
#4TS	#3 AND #2 AND #1

### 2.3 Inclusion criteria

(1) Study type: randomized controlled trial (RCT) either in Chinese or English language, without limitation on the publication time, and including single-blind, double-blind, or non-blind methods. (2) Subjects: Cases with a diagnosis that meets the criteria set by the China Cerebral Palsy Rehabilitation Guidelines (19) and can be diagnosed as CP. (3) Interventions: The treatment group used Jingjin acupuncture therapy in combination with conventional treatment or pure Jingjin acupuncture therapy, and the control group utilized basic treatment. (4) Outcome evaluation indices: total effective rate, MAS Scale, GMFM, FMFM, and the CSS.

### 2.4 Exclusion criteria

(1) Study type: Non-RCT studies. (2) Study population: Non-CP cases and animal experiment-type literature. (3) Interventions: the literature that did not meet the requirements. (4) Efficacy evaluation: the literature whose indicators did not meet the inclusion requirements. (5) Literature with duplicate publications, very low-quality literature, and unclear reporting of original literature were excluded.

### 2.5 Data extraction and risk of bias assessment

The study was independently searched and screened by two evaluators, and then the results were compared, with third-party adjudication in case of disagreement. The results were analyzed by first author, year of publication, number of cases (treatment and control groups), intervention (treatment and control groups), test parameters, duration of treatment, and consistency at baseline. The quality of the literature was assessed using the “Risk of Bias Assessment” tool recommended by the Cochrane Handbook of Systematic Reviews, which assesses the risk of bias for seven items, including generation of randomized sequences, concealment of allocation, blinding of investigators and subjects, blinding of study endpoints, completeness of endpoint data, selective reporting of results, and other biases. Each entry was assessed for risk of bias and categorized as high, low, or uncertain.

### 2.6 Data analysis

The results were analyzed using RevMan 5.4 software. Measurement data were evaluated using the advantages ratio (AR), while continuous variable data were evaluated using mean difference (MD) as the effect size, both expressed with a 95% confidence interval (CI). First, a heterogeneity test was conducted. If *I*^2^ ≤ 50%, the fixed-effects model was used; otherwise, sensitivity analysis and subgroup analysis were conducted first. Following this, either the fixed-effects model or the random-effects model was used directly, depending on the results of the analyses. Publication bias was assessed using a funnel plot, where a symmetrical plot indicated an absence of publication bias, and an asymmetrical plot suggested the presence of publication bias.

## 3 Results

### 3.1 Results of literature detection

**Figure 1:**
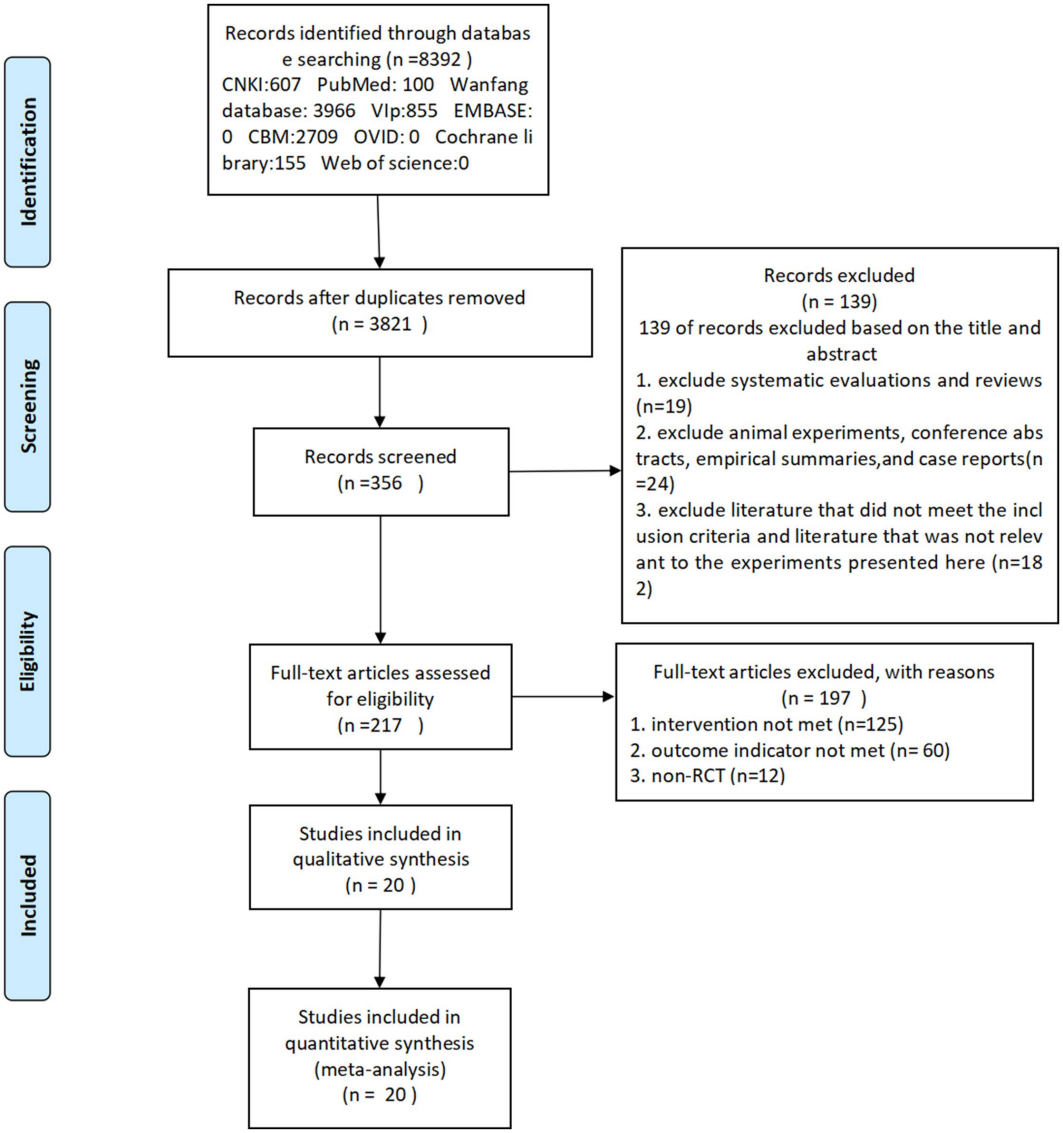
Flowchart of literature search.

A total of 8,239 documents were searched across 9 databases. After checking and reading the titles and abstracts, 356 documents were initially screened. After a full-text screening, documents that did not meet the screening conditions were excluded, resulting in a final inclusion of 20 documents. The process of literature screening is shown in Figure 1.

### 3.2 Basic characteristics of the included studies

All 20 trials were single-center clinical RCTs, with the number of patients in each study ranging from 36 to 137, for a total of 1,453 cases. The included literature met the predefined inclusion and exclusion criteria, and the basic characteristics of all the original studies are shown in Table 2.

**Table 2:** Basic information of the included literature.

First author	Year	Inclusion(T/C)	Article type	Intervention time	Treatment group	Control group	Outcome	Baseline consistency
ZLF (20)	2018	37/38	RCT	48d	JA + RR	RR	①	concordance
LH (21)	2014	30/30	RCT	90d	JA + RR	RR	②④	incoherence
ZY (22)	2016	40/40	RCT	30d	JA + RR	RR	①②⑤	concordance
LQQ (11)	2012	18/18	RCT	90d	JA + RR	RR	②	incoherence
LHP (23)	2019	52/52	RCT	84d	JA + RR	RR	①④	concordance
LW (24)	2021	20/20	RCT	60d	JA + RR	RR	⑤	concordance
RYL (25)	2006	36/19	RCT	60d	JA	RR	④	incoherence
YJR (14)	2016	30/24	RCT	90d	JA + RR	RR	④	incoherence
XWX (16)	2015	28/28	RCT	14d	JA + RR	RR	④	concordance
XWX (26)	2020	71/66	RCT	7d	JA	RR	①④	concordance
TY (17)	2017	20/20	RCT	180d	JA	RR	②④	concordance
YBC (27)	2006	36/34	RCT	30d	JA	RR	④	incoherence
LCG (15)	2014	27/27	RCT	180d	JA + RR	RR	①②	concordance
ZB (18)	2013	45/45	RCT	180d	JA + RR	RR	①②④	concordance
CD (28)	2018	36/36	RCT	90d	JA + RR	RR	①	concordance
QK (12)	2021	30/30	RCT	30d	JA	RR	③④	concordance
LN (29)	2020	50/50	RCT	180d	JA + RR	RR	③	concordance
WJ (30)	2020	45/45	RCT	90d	JA + RR	RR	②	concordance
WHF (31)	2019	30/30	RCT	90d	JA + RR	RR	①②	concordance
GJ (13)	2016	60/60	RCT	180d	JA + RR	RR	①②	concordance

T is the intervention group; C is the control group. ①MAS; ②GMFM; ③FMFM; ④total effective rate; ⑤CSS; JA, Jingjin acupuncture; RR, Routine rehabilitation.

### 3.3 Study risk of bias assessment

**Figure 2:**
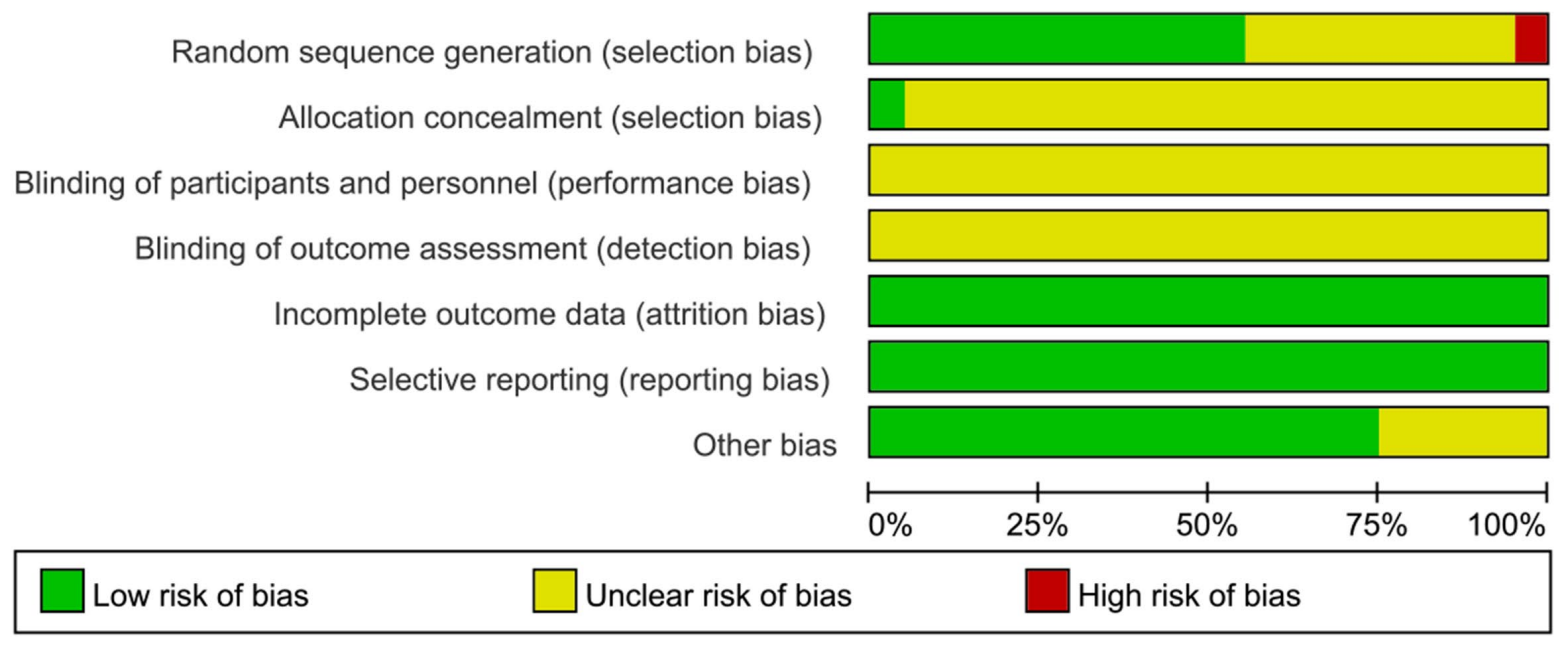
Risk of bias assessment chart.

**Figure 3:**
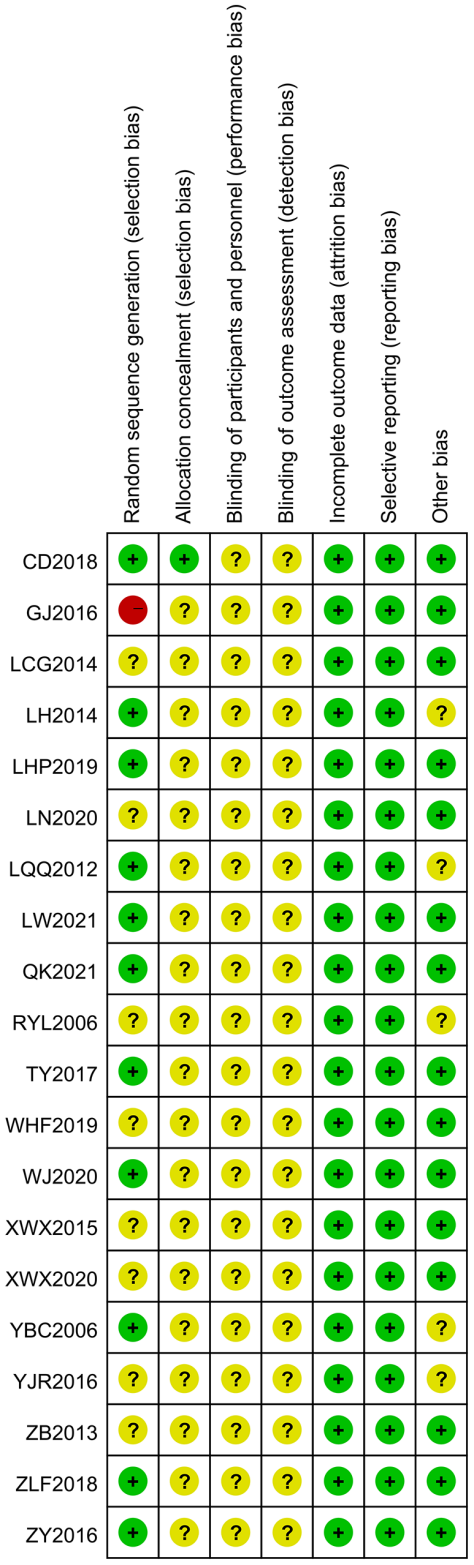
Risk of bias assessment chart.

Twelve of the 20 papers specifically described random sequence generation methods, 8 (24, 30–36) used random number table methods, 2 (23, 27) used systematic randomization, 1 (29) applied random assignment card methods, and 1 (18) used paired design random table methods. In terms of concealment of protocol allocation, only 1 (29) mentioned concealment of protocol allocation by using numbered sealed envelopes, and none of the blinding methods were explicitly applied. A total of 20 papers had complete data without missing, and selective reporting was complete and unbiased. In terms of other biases, five (21, 23, 27, 34, 37) documents did not report whether the baseline data were comparable. A literature risk of bias assessment map was generated by RevMan 5.4 software, as shown in Figures 2, 3.

### 3.4 Analysis of outcome indicators

#### 3.4.1 Overall efficiency

**Figure 4:**
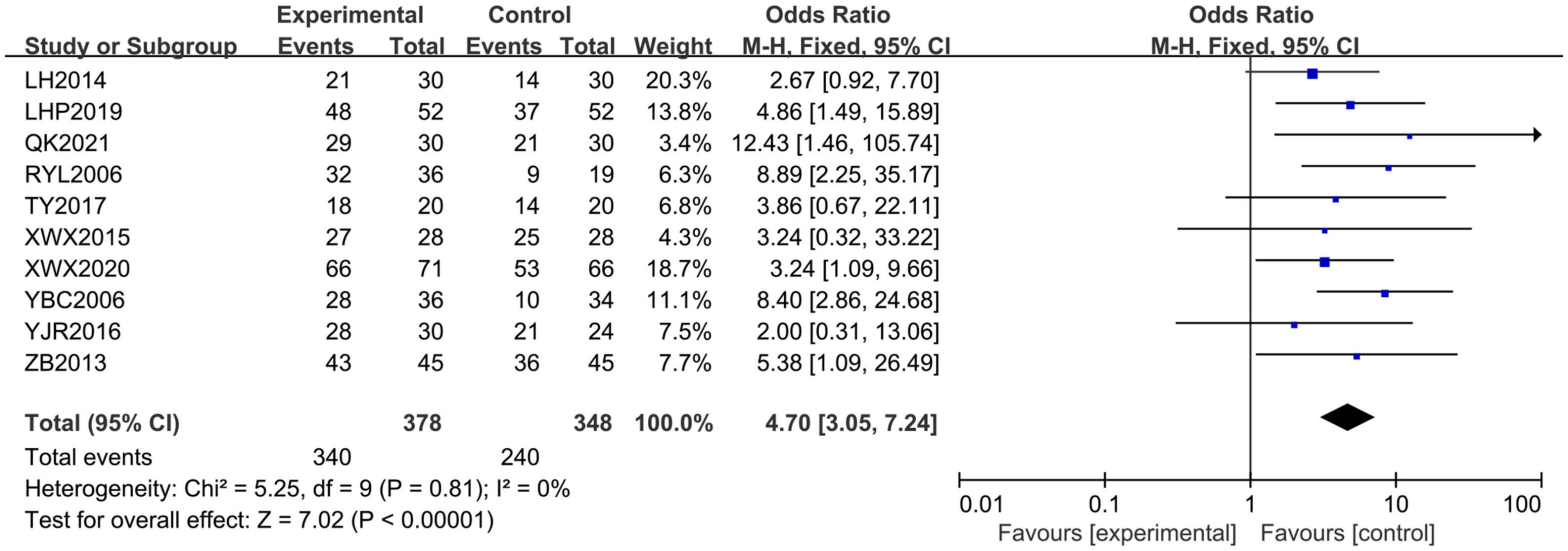
Forest plot of the total effective rate.

Ten (20–23, 26, 27, 30, 31, 34, 35) papers reported overall effectiveness rates. The results of the heterogeneity analysis showed *χ*^2^ = 5.25, *p* = 0.81, and *I*^2^ = 0%, indicating homogeneity among the randomized experiments in terms of statistical quantities. Therefore, a fixed-effects model was used, with an overall OR of 4.70 with a 95% CI of [3.05, 7.24]. The combined-effects quantitative test revealed a statistically significant difference, with *Z* = 7.02 and *p* < 0.00001. In terms of total effective rate, the Jingjin acupuncture therapy was found to be superior to conventional therapy (see Figure 4).

#### 3.4.2 Modified Ashworth spasticity scale

**Figure 5:**
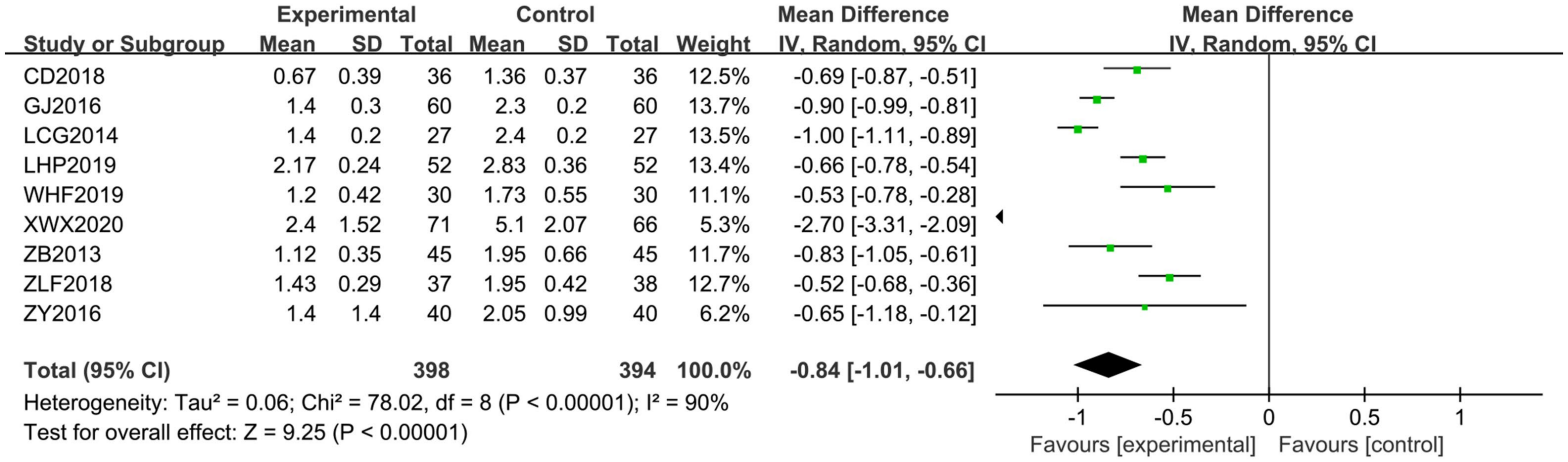
Forest plot of the MAS scale.

**Figure 6:**
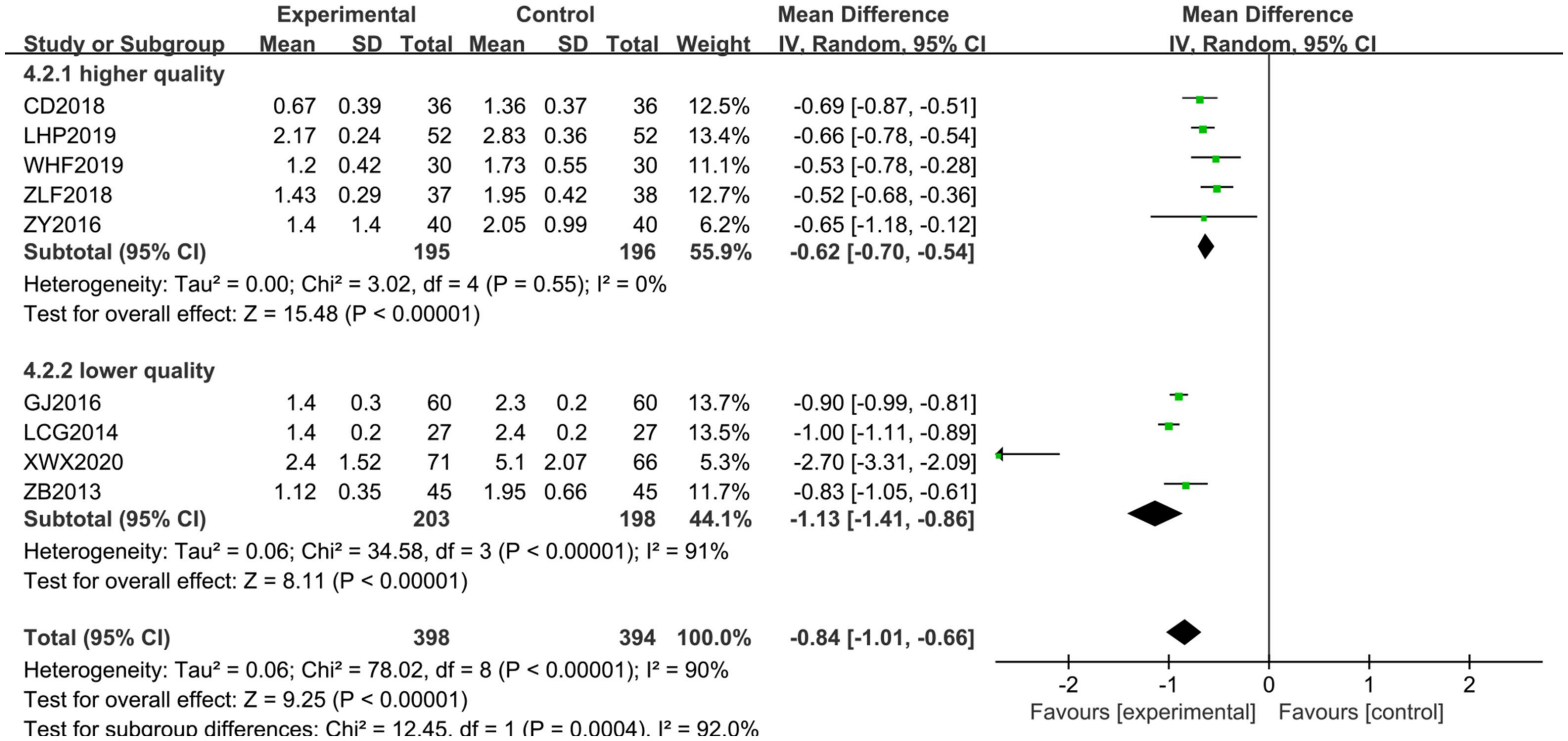
Subgroup analysis of the MAS scale.

Nine papers (20, 22, 25, 28, 29, 32, 35, 36, 38) were scored using the MAS Scale. The heterogeneity of the RCTs of the nine papers was *χ*^2^ = 78.02, *p* < 0.00001, and *I*^2^ = 90%, which was highly heterogeneous and was analyzed using a random-effects model. The total MD was −0.82, with a 95% CI of [−0.87, −0.77]. The combined effect size test showed *Z* = 31.72 and *p* < 0.00001, indicating a statistically significant difference (see Figure 5). To analyze the source of heterogeneity, sensitivity analysis was performed using the literature-by-exclusion method. Even after excluding each literature individually, high heterogeneity persisted. This led to an analysis suggesting potential differences in the quality of the literature. Subsequently, subgroup analyses were performed according to the quality of the literature. There was homogeneity among the high-quality literature included in the studies (*I*^2^ = 0%, *p* = 0.55), and the treatment group had lower scores than the control group; and there was heterogeneity among the studies of the low-quality literature (*I*^2^ = 91%, *p* < 0.00001), with the treatment group scoring lower than the control group, and the Jingjin acupuncture therapy was found to be superior to conventional therapy in terms of the MAS Scale to evaluate the improvement of muscle tone in spastic muscles in post-CP patients (see Figure 6).

#### 3.4.3 Gross motor function scale (GMFM)

**Figure 7:**
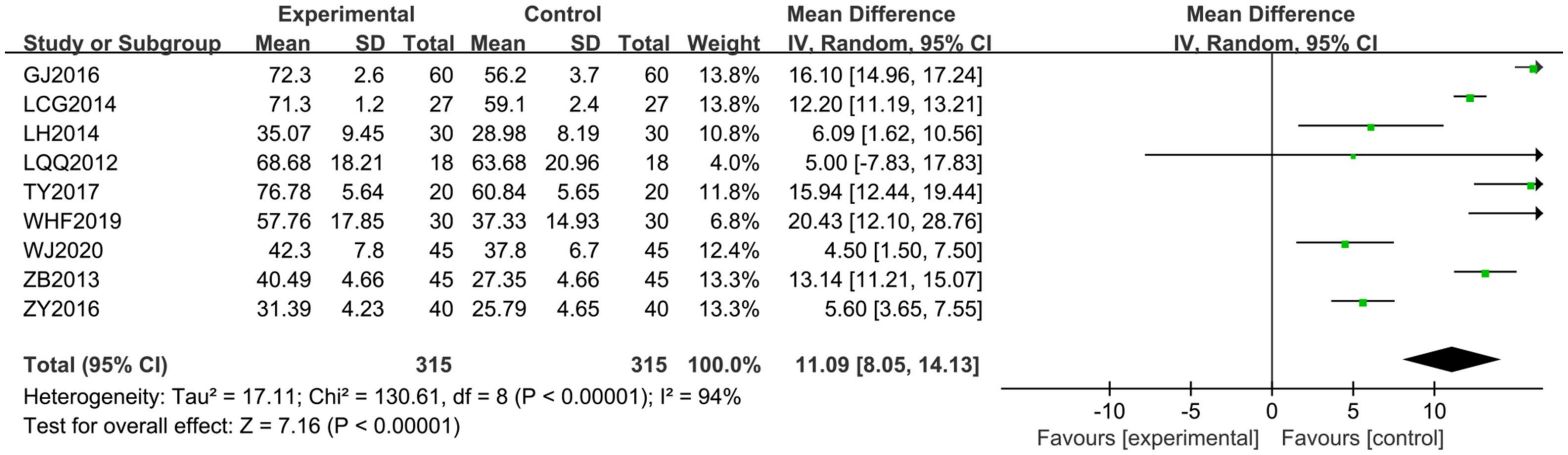
Forest plot of the GMFM scale.

**Figure 8:**
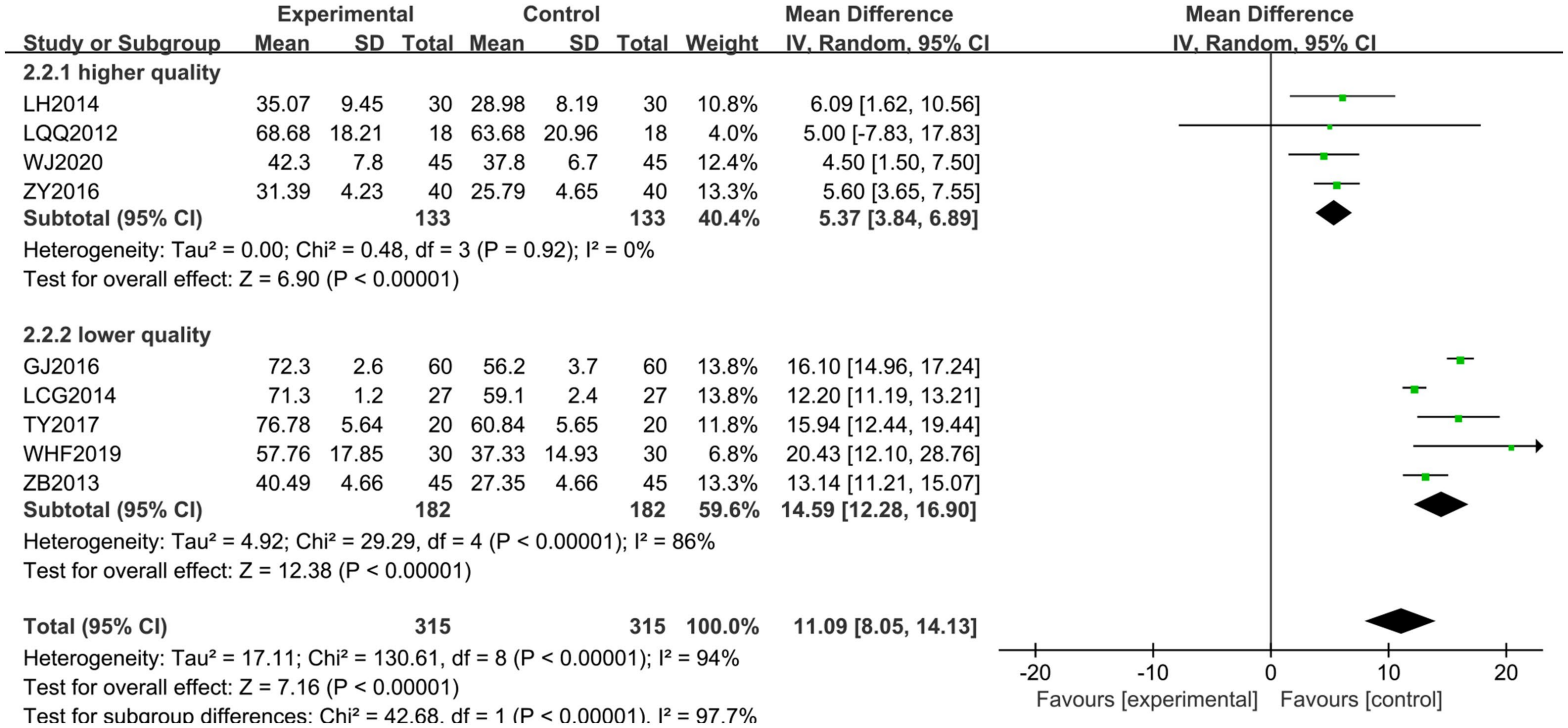
Subgroup analysis of the GMFM scale.

Nine documents (18, 20, 24, 25, 28, 31, 34, 36, 38) were scored using the GMFM. The heterogeneity of the RCTs among the nine documents was *χ*^2^ = 130.61, *p* < 0.00001, and *I*^2^ = 94%, indicating high heterogeneity. Therefore, a random-effects model was used for the analysis. The total MD was 11.09, with a 95% CI of [8.05, 14.13]. The combined effect size test showed a statistically significant difference, with *Z* = 7.16 and *p* < 0.00001 (See Figure 7). To analyze the source of heterogeneity, sensitivity analysis was performed by excluding the literature one by one and excluding any literature. There was still high heterogeneity, indicating that there might be a difference in the quality of the literature, so subgroup analyses were performed according to the quality of the literature. There was homogeneity among the high-quality literature included in the studies (*I*^2^ = 0% and *p* = 0.92), and the score of the treatment group was higher than that of the control group. However, there was heterogeneity among the studies of the low-quality literature (*I*^2^ = 86%, *p* < 0.001), with the treatment group scoring higher than the control group. Jingjin acupuncture therapy was found to be superior to traditional needling in terms of improvement of spastic muscle tone in patients with post-CP as evaluated by gross motor function (see Figure 8).

#### 3.4.4 Fine motor function test scale (FMFM)

**Figure 9:**
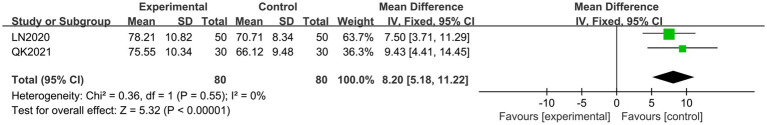
Forest plot of the FMFM scale.

Two papers (30, 39) were scored using the FMFM score. The heterogeneity of the two RCTs was *χ*^2^ = 0.36, *p* = 0.55, and *I*^2^ = 0%, indicating homogeneity. Therefore, a fixed-effects model was used for the analysis. The total MD was 8.20, with a 95% CI of [5.18, 11.22], and the combined effect size test showed a statistically significant difference, with *Z* = 5.32 and *p* < 0.00001. Jingjin acupuncture therapy was superior to traditional acupuncture in terms of fine motor ability evaluation of improvement in muscle tone of spastic muscles in patients after CP (see Figure 9).

#### 3.4.5 Comprehensive spasticity scale (CSS)

**Figure 10:**
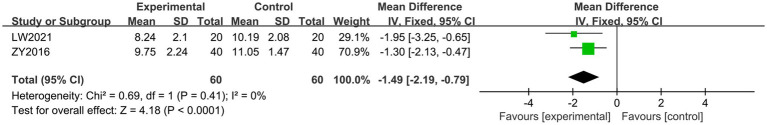
Forest plot of the CSS scale.

Two papers (33, 36) used the CSS score of the affected lower limb. The heterogeneity of the two RCTs was *χ*^2^ = 0.69, *p* = 0.41, and *I*^2^ = 0%, indicating homogeneity. Therefore, a fixed-effects model was used for analysis. The total MD was −1.49, with a 95% CI of [−2.19, −0.79]. The combined effect size test showed a statistically significant difference, with *Z* = 4.18 and *p* < 0.0001. In terms of the CSS score of the affected lower limb before and after treatment to evaluate the improvement of muscle tone of spastic muscles in patients after CP. Jingjin acupuncture therapy was found to be superior to traditional acupuncture (see Figure 10).

### 3.5 Inclusion of literature publication bias

**Figure 11:**
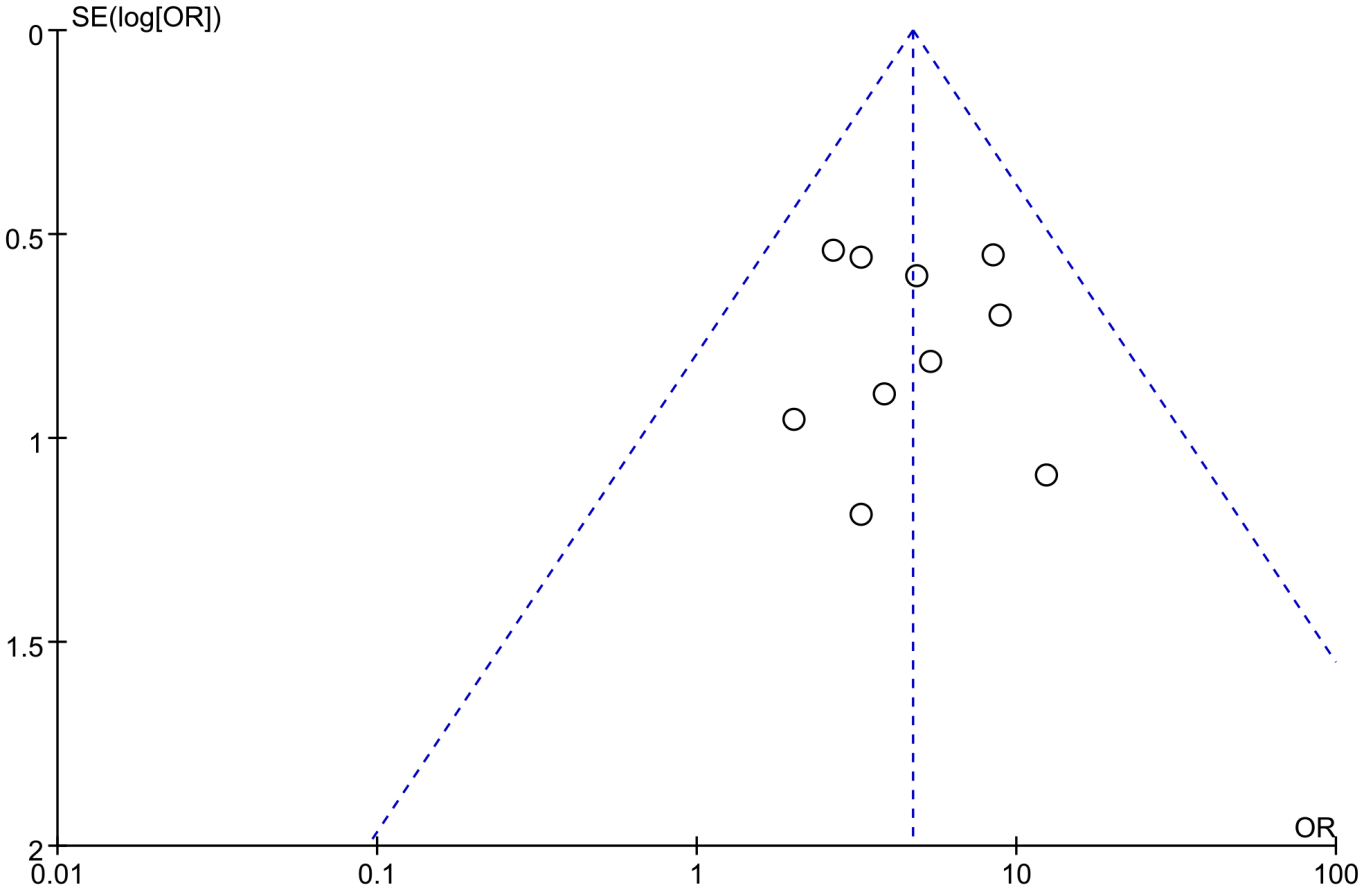
Funnel plot of the total efficiency.

Publication bias was assessed by plotting a funnel plot of the total effectiveness rate of the outcome metrics for the most included studies. The 10 studies were distributed roughly symmetrically around the center line, indicating that publication bias was insignificant and had a negligible effect on the amount of combined effect (see Figure 11).

## 4 Discussion

In this systematic evaluation and meta-analysis, the effects of Jingjin acupuncture on spasticity with CP were investigated. The meta-analysis showed a favorable trend in most of the studies.

Abnormal muscle tone (increased or decreased) is one of the most striking symptoms in patients with CP; it makes movement difficult or even impossible. Nine papers (20, 22, 25, 28, 29, 32, 35, 36, 38) assessed spasticity using the Modified Ashworth Scale, which is a clinical indicator of muscle spasticity in patients with neurological disorders (37). There was a significant difference between the two groups in terms of spasticity after the intervention.

Gross motor function is a frequently measured outcome; almost half of the selected studies applied the GMFM (40) to determine the level of functional skills of the subjects. Nine documents (18, 20, 24, 25, 28, 31, 34, 36, 38) reported positive changes, with all of them showing statistically significant improvements in the GMFM total scores.

The effect of Jingjin acupuncture therapy on the improvement of CP was assessed using not only the GMFM and MAS but also several other methods, including overall effectiveness, FMFM, and CSS. On the one hand, this diversity is advantageous because it has been demonstrated in several ways that Jingjin acupuncture therapy improves spasticity. On the other hand, the fact that the same expected results have been tested in different ways makes statistical analyses challenging. All studies reported positive changes in parameters.

Meta-analyses on Jingjin acupuncture therapy for spastic CP are few in the world, and some researchers have performed some meta-analyses (41, 42) of the efficacy of Jingjin acupuncture versus conventional acupuncture for other disorders and have found that Jingjin acupuncture does have its advantages.

### 4.1 Significance

Spastic CP is divided into spastic quadriplegia, spastic hemiplegia, and spastic diplegia. The site of damage in spastic CP is mainly concentrated in the pyramidal system. When the lesion damage in the brain involves the motor neurons in the cerebral cortex, the motor neurons in the spinal cord lose the constraints of the higher motor neurons (43). This results in the weakening of the downward inhibitory effect and a relative enhancement of the excitatory effect of the peripheral afferent nerves. Consequently, there is a decrease in the excitatory threshold of the detrusor receptors, leading to the stimulation of the muscle shuttles to activate the *α*-motor neurons and the contraction of the extra-shuttle muscles. This phenomenon is manifested as abnormal spasticity of the limbs. Therefore, reducing muscle tone and relieving limb spasticity are essential for treating spastic CP (44).

According to TCM, the pathogenesis of CP is mostly due to the congenital insufficiency of the endowment of the child. This results in a lack of nourishment of the liver, spleen, kidneys, and heart, resulting in a deficiency of qi and blood in these organs. This deficiency further leads to the loss of nourishment of the tendons and veins of the affected child, resulting in bone softness and weakness. Additionally, there is a loss of fullness in the medulla oblongata and lack of mental clarity, which manifests as developmental delays, impairment of limb movement, language disadvantage, and mental retardation. At the same time, dysfunction of the four organs (liver, spleen, kidney, and heart) also leads to long-term insufficiency of qi and blood in the children’s meridians and skin, which is not moistened. This condition, combined with phlegm and stasis blocking the collaterals, prevents qi and blood from running smoothly through the meridians. This creates a vicious circle, making CP an intractable disease (45).

Jingjin acupuncture therapy is based on the principle of “treating with burnt needles and robbing and stabbing, counting with knowledge and losing with pain.” It combines this principle with the theory of Jingjin, using custom-made needles to superficially stab the subcutaneous fascial layer. This process aims to promote the body’s surface Wei qi, soothe and regulate meridians and tendons, and apply treatment to meridian tendons. This approach is a special kind of acupuncture method (46), characterized by its ease of operation and rapid onset of action. It is known for being easy to operate, fast-acting, minimally painful, and highly accepted by patients. In clinical use, compared to ordinary millimeter needling, it is safer and more effective, with better comfort and less resistance to needling in the treatment process (47). Jingjin needling involves a transverse, shallow puncture of the subcutaneous fascia. This technique aims to promote Wei qi, and to dredge and regulate the meridians and tendons (48). Wei qi is the key factor for the effectiveness of Jingjin acupuncture, and the technique of superficial subcutaneous stabbing with Jingjin needles is a means to regulate the operation of Wei qi. Jingjin acupuncture is gentle, and does not cause an increase in muscle tension in children, as it does not elicit a strong feeling of acidity and distension. In the practical application of rehabilitation therapy, we can look for positive reaction points (muscle tension points, muscle fiber nodules, and subcutaneous strips) along the direction of muscle fibers in the lesion area of the child and then insert the needle along the muscle direction, with the needle pointing in the direction of the lesion area.

The results of this study showed that the experimental group exhibited a significantly higher total effective rate of spasticity improvement, GMFM score, and FMFM score compared to the control group. Additionally, the MAS score and CSS score were significantly lower in the experimental group than in the control group, these findings suggest that Jingjin acupuncture therapy can significantly reduce the degree of spasticity in patients with CP, improve their motor function, and enhance their ability to perform daily life activities. These results provide a basis for the argument in favor of using Jingjin acupuncture therapy to treat spasticity in CP patients.

### 4.2 Heterogeneity and potential heterogeneity analysis

The large heterogeneity observed between studies on the MAS and GMFM scales may be due to differences in study quality. Subgroup analyses were performed according to the high and low quality of the literature. While the heterogeneity of the high-quality literature disappeared, it persisted in the lower-quality literature. This suggests an increased likelihood of false-negative and false-positive results regarding significance. This meta-analysis differed in the setting of the control group, which in the 20 studies consisted of routine rehabilitation (18, 23, 28, 29, 31, 35, 36, 38), acupuncture therapy (16, 20), and traditional rehabilitation therapies such as external immobilization in plaster. Conventional rehabilitation was the most frequently used, and although all rehabilitation therapies were utilized, the specific implementation of the course of treatment, location, and means of rehabilitation were not the same. In addition, there are some differences in the treatment group settings in this study. Due to the diversity of the means of Jingjin acupuncture therapy, the treatment measures of the treatment groups in the 20 studies, which included therapies such as needle knife (21, 26, 31), millimeter needle needling (34), and Zhuang medicine picking and stabbing (32), were not completely standardized. There are also fewer studies included in the FMFM and CSS scales, which may increase the probability of bias.

### 4.3 Limitations

First, the 20 studies included in the meta-analysis were all Chinese studies, with no foreign studies, so differences in implementation at home and abroad could not be more fully evaluated. Second, there is a lack of use of the Cochrane-recommended GRADE methodology to assess the quality of evidence. Third, some studies had small overall sample sizes, there were no large samples or multicenter studies, random allocation methods were not described in detail in some studies, and few studies involved allocation concealment or blinded designs. Measurement bias and implementation bias are present, which may exaggerate clinical treatment effects. Fourth, the presence or absence of dropout cases was not mentioned in each study. In addition, most of the studies were not followed up to observe long-term efficacy. Fifth, some studies had unequal sample sizes in the control and trial groups, which may pose a risk of bias. Sixth, some studies were of low quality, and there may be inaccuracies in the interventions or testing indicators during the study. The above shortcomings may have a certain impact on the evaluation results and limit the strength of the argument of this study to a certain extent.

### 4.4 Revelations

Steps to make the treatment more scientific and standardized are as follows: (1) standardizing clinical diagnostic criteria, inclusion criteria, intervention criteria, and efficacy criteria. (2) designing reasonable and scientific, conducting high-quality multi-center, large-sample randomized controlled clinical trials, and paying attention to describing the method of random allocation, allocation concealment, blinding, adverse reactions, and follow-up. (3) studying in-depth the timing, selection, depth, intensity, manipulation, and course of acupuncture points.

## 5 Conclusion

To summarize, Jingjin acupuncture therapy can alleviate the abnormal muscle tone of children with spastic CP, improve the clinical therapeutic effect, and have clinical promotion value in the treatment of dystonia. At the same time, it is hoped that future studies will adhere to more scientific and standardized designs and operations. Improving the quality of the literature will provide more reliable evidence for the clinical treatment of pediatric CP with Jingjin acupuncture therapy.

## Data availability statement

The original contributions presented in the study are included in the article/supplementary material, further inquiries can be directed to the corresponding author.

## Author contributions

XK: Writing – original draft. YH: Conceptualization, Writing – original draft. YZ: Data curation, Writing – original draft. QZ: Formal analysis, Writing – original draft. RG: Funding acquisition, Writing – original draft. JT: Investigation, Writing – original draft. LM: Project administration, Writing – original draft. SC: Methodology, Writing – original draft. XL: Data curation, Writing – original draft. SS: Writing – review & editing, Visualization.
